# Insight in the Chemistry of Laser-Activated Dental Bleaching

**DOI:** 10.1155/2015/650492

**Published:** 2015-03-22

**Authors:** Roeland Jozef Gentil De Moor, Jeroen Verheyen, Andrii Diachuk, Peter Verheyen, Maarten August Meire, Peter Jozef De Coster, Filip Keulemans, Mieke De Bruyne, Laurence James Walsh

**Affiliations:** ^1^Department of Restorative Dentistry and Endodontology, Ghent Dental Laser Centre, Ghent Dental Photonics Research Clustre, Dental School, Ghent University, Ghent University Hospital, De Pintelaan 185-P8, 9000 Ghent, Belgium; ^2^Department of Clinical Neurosciences, John van Geest Centre for Brain Repair and Wellcome Trust-Medical Research Council Stem Cell Institute, University of Cambridge, Cambridge Biosciences Campus, Clifford Allbutt Building, Cambridge CB2 0QH, UK; ^3^SOLA Academy, Bernhard Gottlieb University Clinic of Dentistry, Sensengaße 2A, 1090 Vienna, Austria; ^4^The School of Dentistry, University of Queensland, Clinical Building, Room 216, 200 Turbot Street, Brisbane, QLD 4000, Australia

## Abstract

The use of optical radiation for the activation of bleaching products has not yet been completely elucidated. Laser light is suggested to enhance the oxidizing effect of hydrogen peroxide. Different methods of enhancing hydrogen peroxide based bleaching are possible. They can be classified into six groups: alkaline pH environment, thermal enhancement and photothermal effect, photooxidation effect and direct photobleaching, photolysis effect and photodissociation, Fenton reaction and photocatalysis, and photodynamic effect.

## 1. Definition

The terms “whitening” and “bleaching” are often used interchangeably, which can lead to confusion when interpreting the literature. According to the US Food and Drug Administration (FDA), whitening restores teeth to their natural tooth colour, whereas bleaching makes teeth lighter than their natural colour. In other words, whitening refers to the removal or decolourizing of external stains on the surface of teeth, for example, by means of polishing agents in dentifrices, whereas bleaching is concerned with changing coloured substances within tooth structure (internal or intrinsic stains), for example, using reactive oxygen species (ROS).

## 2. Light Activated Bleaching

The first description of professional bleaching of discoloured teeth was provided by M'Quillen in 1867 [1]. This led in 1895 to the first commercial bleaching product, pyrozone, which was a mixture of five parts of 25% hydrogen peroxide (HP) and one part of diethyl ether [2].

Electromagnetic radiation sources were used for the first time to increase the effectiveness of bleaching in 1937 by Ames, who heated 35% HP [3]. The same approach has been used since the early 1980s, typically in the form of infrared lamps as a heat source to accelerate bleaching using concentrated HP. This approach was effective, but it was also associated with thermal stress, leading to pulp irritation. There were also difficulties in controlling caustic effects from the 35% HP liquid [4].

In 1989, dental bleaching was revolutionized with the advent of nightguard vital bleaching, a technique using a custom-made mouthguard worn at home with a low strength product such as 10% carbamide peroxide, allowing for sustained release of HP [5]. Dental bleaching was no longer reserved for the single discoloured tooth, as now multiple teeth could be whitened.

At the present time, the two most commonly used bleaching procedures are home-based bleaching methods which rely on bleaching gels with low HP concentrations (either present as HP or released from carbamide peroxide) and in-office bleaching using high HP concentrations. In order to reduce the in-office bleaching time, various methods of accelerating the decomposition of HP are used, including chemical (e.g., alkaline pH), physicochemical (e.g., photooxidation), and physical (e.g., heat) techniques. The latter is often described by the term “power bleaching” [6, 7]. Coherent and incoherent radiation sources used to catalyse the bleaching process have included quartz tungsten halogen lamps, plasma arc lamps, mercury vapour lamps, light emitting diodes (LEDs), and lasers of various wavelengths [8–10]. Some proponents of “power bleaching” with intense light sources have claimed that adding various colourants to bleaching gels results in improved absorption of light in the gel and consequent reduced heating of the dental pulp. As well as warming the gel (photothermal effect), colourants can also trigger other photochemical reactions such as photodynamic reactions [11].

## 3. Laser Target Interactions

Laser light can have four possible interactions with the target tissue, depending on its optical properties [12, 13].

1. *Absorption* of laser energy by the intended target: the amount of energy absorbed depends on characteristics such as pigmentation and the presence of light absorbing agents (i.e., chromophores), the laser wavelength used, and the laser emission mode. Light absorption can result in a range of photochemical processes, including photothermal effects (heat emission), fluorescence (light emission), photooxidation (photobleaching), or photodynamic effects.
2. *Transmission* of laser energy through the target: this happens because the laser energy is not absorbed by the target but instead passes harmlessly through it. This effect is highly dependent on the wavelength of the laser used and the target tissue's optical characteristics.
3. *Reflection* of the beam from the surface of the target, with no effect on the target: there are two patterns of reflection.* Specular reflection* is the perfect mirror-like reflection of light from a surface. This is in contrast to* diffuse reflection*, where incoming light is reflected in a broad range of directions. A familiar example of the distinction between specular and diffuse reflection is the finish of gloss paint versus matte paint. Whether the surface is microscopically rough or smooth in proportion to the laser wavelength used has a tremendous impact upon the pattern of reflection.
4. *Scattering* spreads the light into the target across multiple directions, which weakens the intensity of the laser energy at any one point.

For as far as the process of bleaching is concerned, absorption of laser light in the bleaching gel is needed. HP is optically clear which means that its ability to absorb visible light wavelengths is extremely low; however it can absorb ultraviolet, middle infrared, and far infrared light, leading to its breakdown. Without the addition of a colouring agent, one cannot expect HP to absorb visible or near infrared laser light to any great extent. By choosing appropriate chromophores, a range of processes can be triggered. Absorbing energy will produce some localized heating, which increases the production of reactive oxygen species (ROS), in proportion to the elevation of temperature. Caution is needed so that the heat generated in the gel by the absorption of light does not cause general heating of the adjacent tooth to occur, since this would lead to thermal stress at the level of the dental pulp. By choosing different absorbing agents, other photochemical processes such as photodynamic effects can be achieved, leading to even greater levels of ROS. The absorption of light in the gel also reduces the amount of light that can pass through the tooth to reach the dental pulp and in the same light path both the enamel and the dentine [14].

The light absorbing properties of dentine, enamel, and dental pulp all differ from one another, with ultraviolet light and infrared light being well absorbed in the hard tissues, creating a risk of thermal stress to the dental pulp. The least absorbed of the various types of visible light is green light. This optical effect can be exploited in methods which use the effective transmission of visible green light through enamel to target complexes of tetracycline within tooth structure, without heating and/or damage to adjacent tooth structure. The effect of green light on tetracycline can be described as photooxidation or direct photobleaching. Such effects can provide an added value for laser assisted bleaching [14].

## 4. Mechanisms of Tooth Bleaching

Bleaching using ROS (such as HP or derived from HP) is a chemical process that occurs by oxidative decomposition of chromophores, that is, breakdown of these coloured light absorbing organic molecules. Photons (i.e., light particles) are absorbed by the target molecules (chromophores) because of their extended *π*-*π* conjugated system containing delocalized electrons. Conjugation is the overlap of one p-orbital with another across a sigma bond, typically by having alternating single and double chemical bonds. These overlapping p-orbitals allow for a delocalization of *π*-electrons across all the adjacent aligned p-orbitals. The *π*-electrons do not belong to a single bond or atom, but rather to a group of atoms, hence “delocalized.” This leads to the concept of “molecular orbitals” (MOs). The energy difference between the highest occupied molecular orbital (HOMO) and the lowest unoccupied molecular orbital (LUMO) is the “band gap.” This band gap is the amount of energy that is needed to bring a delocalized *π*-electron from HOMO (ground state) to LUMO (excited state). Light with the same energy (i.e., wavelength) as the band gap will be absorbed by the molecule and excite the *π*-electron. Altogether, the MOs that are formed by *π*-*π* conjugated systems lead the absorption spectrum of that molecule, which depends on the chemical structure [15]. Bleaching results from disruption of these *π*-*π* conjugated systems by oxidation (i.e., the loss of electrons) or other chemical reactions. Typically, molecules with ring structures have these opened up into linear forms. This leads to loss of light absorbing properties and consequent discolouration of these molecules.

While HP is used widely in dental bleaching, it should be recognised that many other oxidizing agents (i.e., oxidants) exist which can cause bleaching reactions, with ozone being a common example. All oxidants have a different “bleaching potential” based on their redox potential (*E*°, given in Volts). The stronger this potential is, the more effective the agent will bleach chromophores [16–18]. Listed in increasing order, the values for redox potential are as follows: oxygen (O_2_: +1.229 V); hydroperoxyl radical (HO_2_^∙^: +1.510 V); sodium hypochlorite (NaClO: +1.630 V); hydrogen peroxide (H_2_O_2_: +1.780 V); ozone (O_3_: +2.075); and the hydroxyl radical (HO^∙^: +2.800 V). Due to its high redox potential, hydroxyl radicals are the strongest ROS. Another very potent ROS is singlet oxygen (^1^O_2_); however this cannot be classified as a standard oxidizing ROS because it is able to decompose organic materials in many ways other than oxidation (e.g.,* ene*-type and Diels-Alder reactions). Because of the very high reactivity of hydroxyl radicals and singlet oxygen, these cannot be stored and can only be formed* in situ* as required [16, 19–22].

In terms of the targets for oxidation, as already outlined, in discoloured teeth the coloured stains (chromophores) can be present above the enamel surface or inside the tooth structure itself. Stains on the surface can in most cases be removed easily by professional cleaning, for example, by a prophylaxis using a mild abrasive paste or fine flour of pumice. This is done as a clinical step immediately prior to an in-office bleaching procedure. As well as improving the appearance of teeth by increasing the spectral reflection of light, this external cleaning process removes both saliva and polyphenols (such as tannins) on the tooth surface which can inactivate ROS. Surface stains can also be decolourized, rather than removed, and this mechanism explains much of the cosmetic benefit gained by very low strength (0.5%) HP rinses, paint-on gels, and various oxygen releasing products such as denture cleansers.

For chromophores which reside inside tooth structure, a penetrating action is needed, in the form of either light which is transmitted internally and causes photooxidation (as discussed above for green light) and/or the production of ROS, leading to radicals which penetrate readily through the crystallized structure of enamel and dentine. When considering the selection of agents used for tooth bleaching, they should (i) be strong oxidizers (i.e., have a high redox potential), (ii) generate the types of ROS which are able to diffuse most readily into the dentine, (iii) be noncorrosive, and (iv) be nontoxic. Taking all this into consideration it has been found and repeatedly confirmed that ROS are both potent and safe tooth bleaching agents [18, 19, 23].

## 5. Bleaching with Lasers

The essential elements of laser light that determine its reaction with the target are the wavelength of the radiant energy (nm) emitted by the laser, the power density of the beam (measured in a square centimetre as the unit area—W/cm²), and the temporal characteristics of the beam energy, such as continuous versus pulsed delivery, pulse rate (Hz), and pulse duration. With a pulsed laser, it is more practical to talk about the amount of energy per pulse in Joules (1 J = 1 W/s), rather than the average output power in watts. Other useful measures are energy density (J/cm²) and the amount of energy per unit area (fluence). In addition to these factors, there are several other variables that relate to differences in how the laser energy is delivered, such as contact versus noncontact delivery mode, focused versus unfocused beams, and beam diameter.

Next to the wavelength of the laser, the mechanism of action of the laser is affected greatly by the power of the radiation delivered and the mode of operation. The type of mode influences the dynamics of heating of the target; that is, accumulated heating with continuous wave mode is higher than that with pulsed mode. At low irradiances and/or energies, laser-tissue interactions are either purely optical or a combination of optical effects, photochemical effects, and photobiostimulation. When laser power or pulse energy is increased, photothermal interactions begin to dominate. Pulsed lasers can create very high power densities within a very short time, leading to photoablation [24–26]. Such effects are not desirable in a bleaching gel.

Combining these various points, when choosing a laser wavelength to enhance bleaching efficacy it is of the utmost importance to consider the extent to which light “absorption” (which is both wavelength and target dependant) is needed and when it occurs how much of the laser energy will be converted into heat. The absorption of photons will influence the temperature rise which occurs within the bleaching product, the dental hard tissues, and/or pulp tissues. There must be a careful alignment between the laser and the characteristics of the gel, that is, the presence of additives which affect the absorption spectrum and colour. The thickness of the gel and its pH also have to be taken into account; the latter because the pH influences the patterns of radicals which are generated.

## 6. Enhancing Hydrogen Peroxide Based Bleaching

In general, lasers can enhance bleaching by photooxidation of coloured molecules in the teeth or by interaction with components of the bleaching gel, through photochemical reactions. Such effects will be affected by pH, temperature, light, and the presence of catalysts. The different methods of enhancing hydrogen peroxide based bleaching can be classified into six groups.

### 6.1. Alkaline pH Environment

Hydrogen peroxide is always stored at low/acidic pH. At pH values above 7 (alkaline), HP becomes much more reactive and has a very short shelf life. This is due to the loss of a hydrogen atom (H^+^), yielding hydroperoxyl anion (HO_2_^−^), not to be confused with the hydroperoxyl radical (HO_2_^∙^). In this state not only does HP become a safer tooth bleaching product (since acidic products demineralise and damage enamel), but also it will enhance the bleaching efficiency because of the higher reactivity of hydroperoxyl anions. This higher reactivity can be understood by (i) closer association of the anion with positively charged chromophores and (ii) additional reactions (e.g., Dakin reaction and addition to quinone) whereby the anion can disrupt chromophores. HP is most effective for bleaching at pH values between 9.5 and 10.8. Because of its inherent instability at elevated pH, HP is typically mixed with an alkalinizing agent immediately prior to its application in the mouth, in order to obtain this type of bleaching enhancement [16, 20, 27].

### 6.2. Photochemistry

When a molecule is irradiated by photons whose energies just correspond to the difference in energy between the ground state and some higher or excited state of the molecule, the photons are absorbed and the molecule is raised to a higher energy level. Spectra arise when molecules absorb photons of specific energy. When a molecule absorbs light/photons, it will enter a singlet excited state (electron in a higher energy state). The molecule can take up more light/photons, forcing the molecule in a triplet excited state (change in electron spin of the excited electron by intersystem crossing). The excess of energy is lost by heat emission (vibration) or by photon emission (fluorescence for singlet excited molecules and phosphorescence for triplet excited molecules). Furthermore, when in singlet or triplet excited state some molecules are sensitive to certain chemical reactions (disruptions or creation of certain chemical bonds, both intra- and intermolecular) that were not possible in their ground state. All this together is the topic of photochemistry. The following photochemical reactions can be beneficial for enhancement of hydrogen peroxide based bleaching [28, 29].

#### 6.2.1. Thermal Enhancement and Photothermal Effect

Electric heating devices, special designed lamps, and lasers can be used to heat the bleaching gel. This will enhance the bleaching efficiency simply because chemical reactions happen faster at elevated temperature. A 10°C temperature rise can speed hydrogen peroxide decomposition by a factor of 2.2 [26]. Additionally, an increase in heat will allow better peroxide penetration into dental structures [30]. Lamps can produce heat because of inefficient conversion of electric current to heat (e.g., tungsten wires in lamps produce heat). A more direct approach is when “cold” lamps and lasers induce a temperature rise in the target substrate by the photothermal effect. Light will be absorbed by molecules inside the bleaching gel or tooth structures, leading to molecular vibration and consequent heating [30]. The condition however is that the light is absorbed by the molecules, which depends on the absorption spectrum of the molecule and the emission spectrum of the lamp or laser. Problematic with this type of enhancement is that heat can lead to dehydration of the enamel and to irreversible damage of the pulp [20, 26, 31].

#### 6.2.2. Photooxidation Effect and Direct Photobleaching

When certain molecules enter their triplet excited state, they are prone to lose an electron; that is, they become oxidized. This process is called photooxidation. When this happens to chromophores, their *π*-*π* conjugated systems are disrupted and they are consequently bleached, that is, direct photobleaching. In case of tooth bleaching the light has to be able to penetrate the tooth structures (transmission). UV has very good photobleaching properties but does not penetrate well through teeth and causes soft tissue damage, burns, and pulp heating. Green light is optimal for photobleaching since it is not absorbed by water or hydroxylapatite. It will thus nicely penetrate the tooth structure where it can remove chromophores that absorb green light. Because of the high light density, laser light is more efficient in photobleaching than nonlaser light [26, 29, 32–35].

#### 6.2.3. Photolysis Effect

The process of photolysis or photodissociation is one where light is absorbed by a molecule trigging the disruption of a chemical bond and the breakdown of that molecule. In this regard, HP can absorb UV light causing it to break up into two hydroxyl radicals (HO^∙^). Because HO^∙^ is a much more potent bleaching agent, this will enhance the bleaching efficiency. However, as mentioned before, caution has to be taken when using UV light [29, 36, 37].

#### 6.2.4. Fenton Reaction and Photocatalysis

Iron ions (Fe^2+/3+^) can catalyze the production of hydroxyl radicals (HO^∙^) and hydroperoxyl radicals (HO_2_^∙^) out of HP by continuous oxidation and reduction. This reaction is well known as the Fenton reaction. The formed HO^∙^ will boost the tooth bleaching. The downside however is that equimolar amounts of HO_2_^∙^ (weaker than HP) are also formed and that the breakdown of HO^∙^ is also catalyzed by the iron ions. Photocatalysis can circumvent this problem. Fe^3+^ activated with UV light will catalyze the formation of more HO^∙^ out of water. This process is called the photo-Fenton reaction. Of course, in this case HO^∙^ will also be formed due to photolysis of HP. Again, using UV light is not advisable in oral applications [29, 36, 37].

#### 6.2.5. Photodynamic Effect

This technique is based on the photodynamic therapy (PDT) where photosensitive dyes are excited by light. In their triplet excited state, they can donate highly energetic electrons and reduce O_2_ and H_2_O_2_ forming H_2_O_2_, HO_2_^∙^ and HO^∙^. Furthermore, during intersystem crossing (from singlet to triplet excitement) the dyes will form singlet oxygen (^1^O_2_) by spin-orbit coupling with triplet oxygen (^3^O_2_), the common form of molecular oxygen. Altogether, photodynamic bleaching enhancement enables the formation of the two most potent bleaching agents, that is, hydroxyl radical and singlet oxygen, and is thus a very powerful enhancement [16, 21, 22, 29, 32, 38].
